# In Situ Monitoring of Bacteria under Antimicrobial Stress Using ^31^P Solid-State NMR

**DOI:** 10.3390/ijms20010181

**Published:** 2019-01-06

**Authors:** Sarah A. Overall, Shiying Zhu, Eric Hanssen, Frances Separovic, Marc-Antoine Sani

**Affiliations:** 1 School of Chemistry, Bio21 Institute, University of Melbourne, Victoria 3010, Australia; soverall@ucsc.edu (S.A.O.); shiyingz2@student.unimelb.edu.au (S.Z.); 2 Chemistry & Biochemistry Department, UC Santa Cruz, CA 95064, USA; 3 Advanced Microscopy Facility and Department of Biochemistry & Molecular Biology, Bio21 Institute, University of Melbourne, Victoria 3010, Australia; ehanssen@unimelb.edu.au

**Keywords:** antimicrobial peptide, live cell, solid-state NMR, DNA, lipid membrane

## Abstract

In-cell NMR offers great insight into the characterization of the effect of toxins and antimicrobial peptides on intact cells. However, the complexity of intact live cells remains a significant challenge for the analysis of the effect these agents have on different cellular components. Here we show that ^31^P solid-state NMR can be used to quantitatively characterize the dynamic behaviour of DNA within intact live bacteria. Lipids were also identified and monitored, although ^31^P dynamic filtering methods indicated a range of dynamic states for phospholipid headgroups. We demonstrate the usefulness of this methodology for monitoring the activity of the antibiotic ampicillin and the antimicrobial peptide (AMP) maculatin 1.1 (Mac1.1) against Gram-negative bacteria. Perturbations in the dynamic behaviour of DNA were observed in treated cells, which indicated additional mechanisms of action for the AMP Mac1.1 not previously reported. This work highlights the value of ^31^P in-cell solid-state NMR as a tool for assessing the antimicrobial activity of antibiotics and AMPs in bacterial cells.

## 1. Introduction

The emergence of bacterial strains resistant to many important front line antibiotics has fuelled the urgency for which new efficacious antimicrobial agents are being developed. Antimicrobial peptides (AMPs) are produced by many organisms to protect against infections and are an attractive class of antimicrobial agent due to their low cytotoxicity and low occurrence of bacterial resistance [1,2]. AMPs are typically characterized by their cationic charge and amphipathic structure [3] conferring greater activity towards negatively charged membranes, typical of bacteria, while neutral membranes, characteristic of eukaryotes, are less affected [4,5], thus, conferring specificity towards bacterial membranes. The targeted disruption of bacterial membranes by AMPs can occur by several mechanisms from pore formation to detergent like solubilization of membranes [6,7,8]. However, it is unclear what the contribution of membrane disruption is to the bactericidal properties of AMPs. The complex architecture of the bacterial membrane could modulate the mode of action of AMPs in situ, altering the threshold of inhibitory concentrations. Thus, there are still unknowns in understanding how AMPs interact with bacteria and, in particular, how bacteria respond to AMP activity in situ.

Maculatin 1.1 (Mac1.1) is an AMP derived from the skin secretions of the Australian tree frog, *Litoria genimaculata.* Mac1.1 is a typical cationic AMP showing low µM activity against Gram-positive bacteria and moderate activity against Gram-negative bacteria [9,10]. It is proposed to act via a pore-forming mechanism [11]; however, the discrepancy in threshold concentration between bacterial species was recently hypothesized as a consequence of bacterial responses to AMP induced stress instead of peptide activity alone [12]. Activity of AMPs against non-membrane targets have also been documented for other AMPs, displaying direct interaction with bacterial DNA, inhibition of protein synthesis and destabilization of the cell wall [13]. These effects often synergize with membrane permeabilization properties and so understanding how AMPs interact with various cellular components is critical for the rational design of synergistic AMP therapies. Reproducing the complex double membrane structure of Gram-negative bacteria in a model system is challenging given the differential lipid composition of the inner and outer membranes separated by peptidoglycan. To address this, we present an initial in-cell portrayal of intact and viable *E. coli* bacteria using ^31^P solid-state NMR spectroscopy. In-cell NMR is a fast-developing method to investigate the structure and function of biomolecules in their native environment [14,15]. We have applied this approach to study the effect of the well-characterized antibiotic ampicillin and the AMP Mac1.1 on *E. coli* bacteria. We demonstrate the feasibility of using ^31^P NMR to identify nucleic acids (NAs), i.e., DNA and RNA, and lipid components in intact bacteria. Detailed characterization of NA phosphodiester bond dynamics was obtained with only a qualitative assessment of lipid structure and dynamics due to signal overlap and a broad dynamic range. Using this methodology, we show that ampicillin and Mac1.1 have significant effects on the dynamics of NAs resulting in increased motional averaging of the NA signal. These effects could be related to reactive oxygen species (ROS) mediated stress as the presence of thiourea, a ROS scavenger, could partly ameliorate the effects of ampicillin on the NA spectrum. 

## 2. Results

### 2.1. Solid-State ^31^P NMR Experiments on Intact E. coli Bacteria

**Figure 1:**
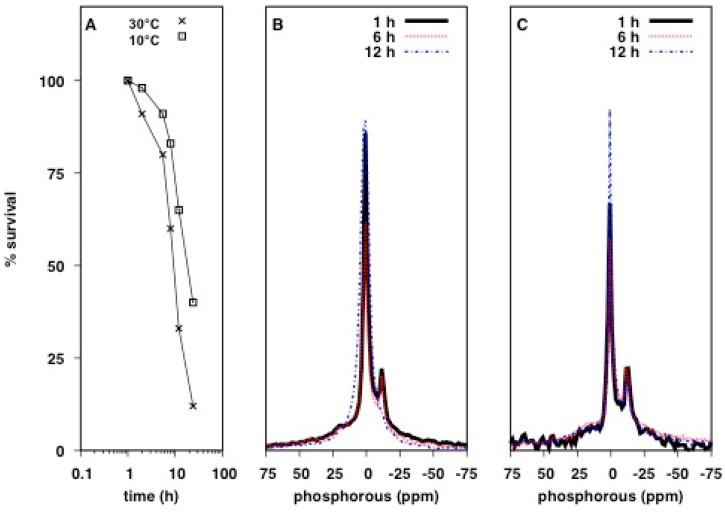
Effect of temperature on bacterial survival over the course of a ^31^P static NMR experiment: (**A**) Bacterial survival over the course of an NMR experiment. 500 mL of *E. coli* culture, grown to an OD_600_ of 0.5 was packed into a 4 mm zirconia MAS rotor as a cell slurry. Colony forming units were determined by taking 5 µL of cell slurry and performing serial dilutions at each time point, followed by plating onto LB agar and enumerating the number of colonies. Data is from a single experiment with CFU dilutions performed in triplicate. Error bars indicate the standard error of the mean per triplicate. (**B**) ^31^P direct excitation spectra of the same samples measured in A at 30 °C; and (**C**) at 10 °C. Spectra were taken 1 h (black solid line), 6 h (red dotted line) and 12 h (blue dashed line) after packing.

Phosphorous containing molecules provide useful markers for understanding how AMPs interact with bacteria as many biomolecules contain phosphorus, particularly, phospholipids and DNA. Utilizing ^31^P solid-state NMR, we investigated the effect of antimicrobial agents on live *E. coli*. Given the use of small NMR rotors holding less than 100 µL of sample, hydration, oxygen availability and nutrient supply could severely limit the sample integrity and, therefore, data quality over the course of an experiment. To determine the length of time in which live cell samples are sufficiently stable for NMR analysis, we assessed bacterial survival at different time points during an NMR experiment at 30 and 10 °C under static condition, i.e., without magic angle spinning (MAS). Samples were prepared by growing bacteria to an OD_600_ of 0.5, media components were removed by washing in isosmotic salt buffer prior to packing the cell slurry into a 4 mm zirconia MAS rotor and immediately acquiring NMR spectra. Cell survival was quantified by the number of colony forming units (CFUs) in an aliquot of cells taken from the rotor at various time points. Bacterial viability was remarkably stable over a 4 h period given the high cell density (>10^10^ CFU/mL). After 12 h the number of CFU decreased by 70% at 30 °C while cell survival was greatly improved by maintaining the sample at 10 °C, in which CFU declined by only 33% over the same period (Figure 1A). ^31^P static spectra collected at 10 and 30 °C showed a superposition of lineshapes, indicative of a wide range of ^31^P environments (Figure 1B,C). After 12 h at 30 °C there was a significant reduction in spectral quality, concomitantly with reduced cell survival (Figure 1A,B). Most notably the intensity of the peak at −11 ppm disappeared and most features merged into a broad isotropic signal, indicative of fast molecular reorientation on the ^31^P NMR time scale, suggesting a significant loss of cellular integrity and hence sample degradation. However at 10 °C, no drastic change was observed, even after 12 h, and so subsequent experiments were performed for up to 6 h at 10 °C.

### 2.2. Qualitative Analysis of ^31^P Lineshapes in Intact Bacteria

**Figure 2:**
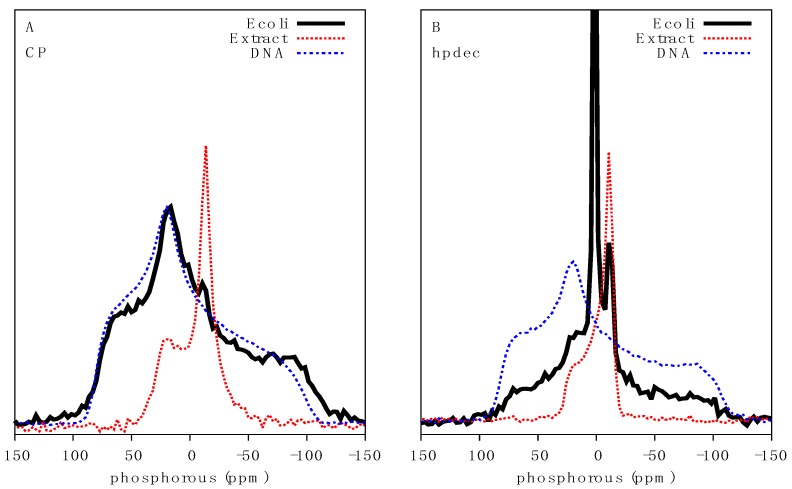
Characterization of bacterial DNA by ^31^P solid-state NMR: (**A**) ^31^P CP spectra using a contact time of 1.5 ms; and (**B**) Direct ^31^P excitation spectra of *E. coli* (black solid line), MLVs made of *E. coli* total lipid extract (red dotted line) and dry genomic DNA (blue dash-dotted line). All spectra were acquired at 10 °C.

In order to gain meaningful insights into the effect of antimicrobial agents on the molecular architecture of intact bacteria, we assessed whether different phosphorus containing biomolecules could be distinguished from one another in live bacteria by ^31^P solid-state NMR. The static ^31^P lineshape is defined by the width of the powder pattern due to the chemical shift anisotropy (CSA, defined using the Haeberlen convention: δ_zz_–δ_iso_) and asymmetry parameter eta (η), which are modulated by the molecular environments and molecular dynamics of the ^31^P tensor. The use of ^1^H to ^31^P cross-polarization (^31^P CP) selectively filters out ^31^P signals of fast rotating molecules and depends on the contact time. Using a relatively short contact time of 1.5 ms, a very broad ^31^P signal with a span of ca. 230 ppm was observed in bacteria (Figure 2A). This is typical of solid or rigid molecules with little motional averaging of the ^31^P tensor. The immobilized nature of the signal identified was surprising considering the cells analyzed were well above freezing temperature and were not only intact but also fully hydrated. We postulated that NAs would be the most likely biomolecules to maintain a rigid-like structure, particularly in the form of condensed genomic DNA or large mRNA that can reach megaDalton size [16]. The distinction between DNA and RNA cannot be achieved under static NMR conditions and, since the DNA/RNA mass ratio in *E. coli* varies from ca. 1:3 at early growth phase to ca. 1:15 at late growth phase [17], the overall NA signal was used.

Comparison of ^31^P CP spectra of purified DNA with that of bacteria revealed identical lineshapes and a comparable chemical shift span or powder pattern of ca. 200 ppm, indicating DNA could be the primary source of this signal. Furthermore, ^31^P CP analysis of MLVs composed of *E. coli* total lipid extract, providing an expected ^31^P static lineshape for phospholipids, showed a chemical shift span of ca. 54 ppm (Figure 2A), typical of a gel phase lipid bilayer. Interestingly, the peak intensity at −11 ppm, indicative of fast-axially reorienting lipids, was also visible by CP, indicating that lipids can be detected in this dynamic range, although the dominance of the NA signal as this contact time precludes analysis. Together the data strongly indicates that broad ^31^P signals in live cells arise due to NAs.

Analysis of direct excitation ^31^P spectra of bacteria, which detects all ^31^P signals irrespective of molecular motion, revealed a convolution of several powder pattern lineshapes (Figure 2B). An intense isotropic signal centered at 1.8 ppm indicates the presence of fast re-orienting ^31^P containing molecules and likely arises from a wide range of molecules from phosphorylated soluble proteins, ATP, ADP and inorganic phosphate species. This component accounts for 38% of the spectral area. The underlying rigid NA signal is also visible, albeit significantly reduced relative to the intensity of isotropic ^31^P dominating the spectrum. A clear powder pattern indicative of a lipid bilayer was also visible as recapitulated by the lipid extracts, typical of a fluid phase phospholipid bilayer (Figure 2B). The outer and inner lipid membranes, which are mainly composed of lipopolysaccharide, phosphatidylethanolamine and phosphatidylglycerol, were difficult to distinguish due to the complexity of the sample. An attempt to deconvolute the ^31^P spectrum of *E. coli* was made using a three-component system accounting for NA, lipid and an isotropic component. Initial CSA and η values already described for dry DNA and lipid extracts were used as component estimates. Lineshape analysis, using the TopSpin solid lineshape fitting tool, of *E. coli* CP spectra gave a CSA of −109 ppm with an η value of 0.52 and an isotropic chemical shift at 1.28 ppm. Lineshape analysis of the fluid phase lipid component of direct excitation spectra yielded a CSA of 25.2 ppm (overall span of ~45 ppm), η value of 0.12 and isotropic chemical shift of −0.38 ppm, while the isotropic component of the spectra showed a chemical shift of 1.6 ppm. Overall, the fit is in good agreement with the values determined for purified DNA and lipid extract.

### 2.3. Characterisation of the Effects of Antimicrobial Agents on Bacteria

**Figure 3:**
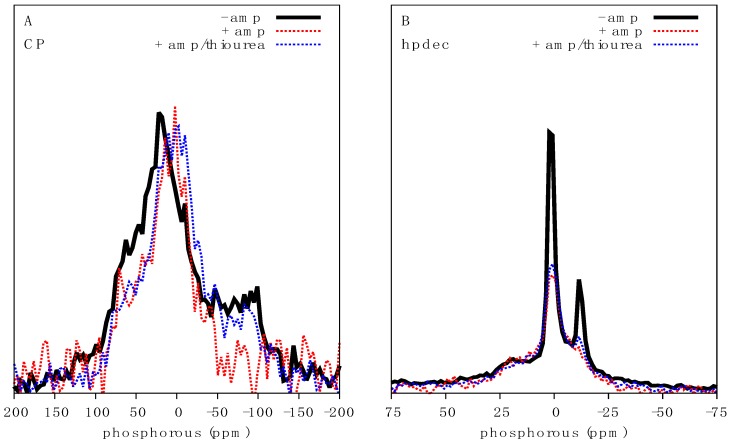
Characterization of the effect of ampicillin on *E. coli*: Bacteria grown to OD_600_ = 0.5 were treated with 20 µg/mL ampicillin for 1 h prior to NMR analysis. (**A**) ^31^P CP spectra at 1.5 ms contact time, and (**B**) ^31^P direct excitation spectra of *E. coli* (black solid line), ampicillin treated *E. coli* (red dotted line) and ampicillin and thiourea treated *E. coli* (blue dotteded line). Spectra have been scaled to facilitate lineshape comparisons.

In order to gain a sense for how AMPs might affect the NMR spectrum of intact bacteria, we first assessed the effects of the well characterized antibiotic ampicillin, whose mechanism of action and cellular effects are well known, including inhibition of cell wall synthesis and damage by reactive oxygen species (ROS) [18,19]. We treated 500 mL *E. coli* cultures with 20 µg/mL ampicillin (minimum inhibition concentration (MIC) of 4 µg/mL against *E. coli* [20]) for 1 h prior to preparation for NMR spectroscopy. Analysis of intensity normalized CP spectra shows a clear reduction in the chemical shift span of the NA signal in ampicillin treated *E. coli*, decreasing from ca. 225 to ca. 150 ppm (Figure 3A). The increased noise in the spectrum is also indicative of a significant reduction in absolute signal intensity observed in ampicillin treated cultures due to cell growth inhibition and cell death. The region of greatest intensity also shifted from 20 to 1 ppm indicating a reduction in tensor asymmetry and increased motional averaging. In the direct excitation spectra, the intensity at −11 ppm due to axially reorienting lipids was significantly reduced (Figure 3B) consistent with membrane perturbations. As ampicillin is not known to directly interact with bacterial DNA, we hypothesized that the reduction in NA chemical shift span could be related to stress induced ROS production [19], possibly damaging NAs through the breaking of phosphodiester bonds resulting in increased ^31^P motion and thus a reduction in linewidth (Figure 3A). To test this we cultured *E. coli* in the presence of ampicillin and thiourea, a powerful ROS scavenger. Provision of thiourea slightly ameliorated the reduction in chemical shift span of ^31^P CP spectra and direct excitation spectra (Figure 3). Signals indicative of fast-axially reorienting lipids (signal at −11 ppm) were only slightly recovered in the presence of thiourea, indicating that thiourea treatment could not completely perturb or negate the activity of ampicillin. 

**Figure 4:**
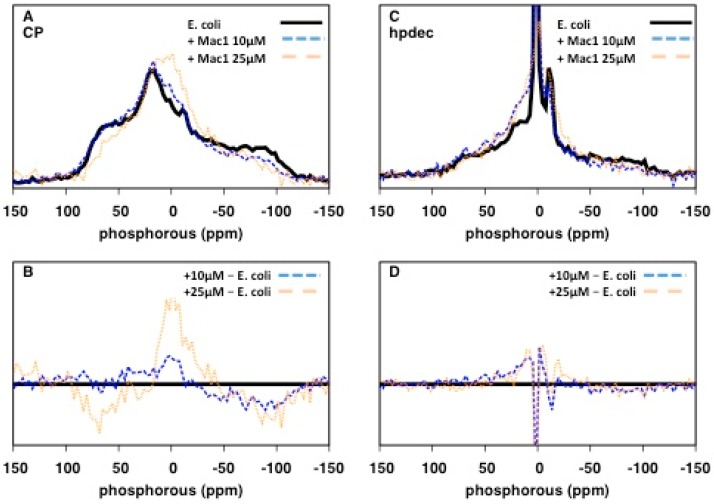
Mac1.1 perturbs NA packing: (**A**) ^31^P 1.5 ms contact time CP spectra; and (**C**) ^31^P direct excitation spectra of *E. coli* (black solid line), in the presence of 10 μM Mac1.1 (blue dashed line) and 25 μM Mac1.1 (orange dotted line). (**B**) Difference between CP spectra, and (**D**) difference between direct excitation spectra of *E. coli* and in the presence of 10 μM Mac1.1 (blue dashed line) or in the presence of 25 μM Mac1.1 (orange dotted line).

We next assessed the impact of the AMP, Mac1.1 on *E. coli* by ^31^P solid-state NMR. *E. coli* cultures were treated with sub-MIC concentrations of Mac1.1 (MIC of order 100 µg/mL or greater against *E. coli*, [12]) for 30 min prior to NMR analysis. At 10 µM Mac1.1, a loss of signal intensity at the edges of the ^31^P CP spectrum was observed (Figure 4A,B). Increasing the amount of Mac1.1 to 25 µM intensified the lineshape alterations. The results indicate a change in NA dynamics with an increase in the proportion of molecules undergoing fast reorientation as observed following ampicillin treatment. Direct excitation ^31^P spectra showed a similar increase in fast-reorienting ^31^P molecules significantly reducing the signal intensity of axially reorienting lipids, indicative of membrane disruption (Figure 4C,D).

**Figure 5:**
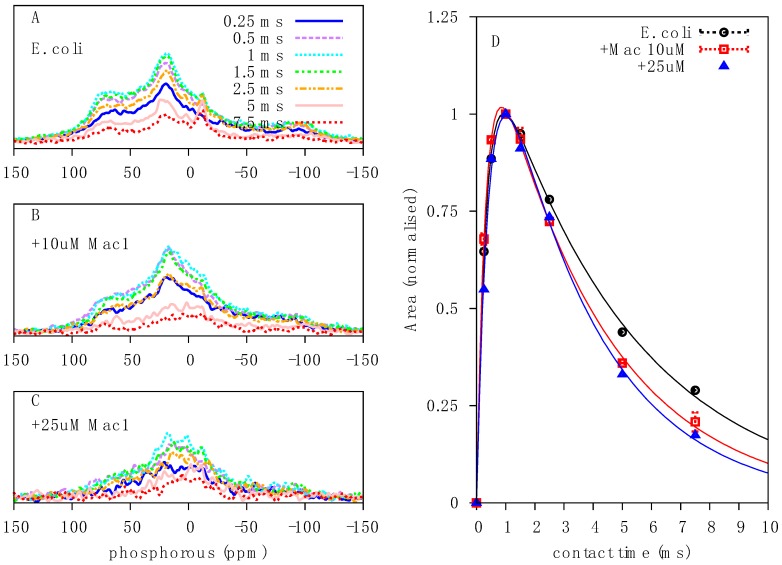
Mac1.1 increased μs motions in NAs: Contact time array in ^31^P CP spectra of: (**A**) peptide-free *E. coli*; (**B**) in the presence of 10 μM Mac1.1; and (**C**) in the presence of 25 μM Mac1.1. The contact times used were (solid blue) 0.25 ms, (dashed purple) 0.5 ms, (dotted cyan) 1 ms, (dash-dotted green) 1.5 ms, (dash-dotted-dotted gold) 2.5 ms, (short-dotted pink) 5 ms and (short-dashed red) 7.5 ms. (**D**) Integral versus contact time plot with fit according to Equation (1) of (circle red line) peptide-free *E. coli*, (square cyan) in the presence of 10 μM Mac1.1 and (triangle blue) in the presence of 25 μM Mac1.1.

Quantitative analysis of CP spectra with various contact times revealed a reduction in T_1ρ_ values following Mac1.1 treatment, dropping from 4.8 to 3.3 ms (Figure 5D), indicative of increased molecular motion in the μs regime and therefore a reduction in molecular order and stability, consistent with the qualitative changes observed in the lineshape and chemical shift span. Given the similarities between Mac1.1 and ampicillin treatment on ^31^P spectra of NAs, we similarly treated *E. coli* with Mac1.1 in the presence of thiourea to determine the contribution of ROS damage to Mac1.1 mediated effects on the spectra. However, rather than being protective, thiourea treatment caused catastrophic cell death when combined with Mac1.1 precluding any analysis due to almost complete loss of signal from the death of the cell culture. 

**Figure 6:**
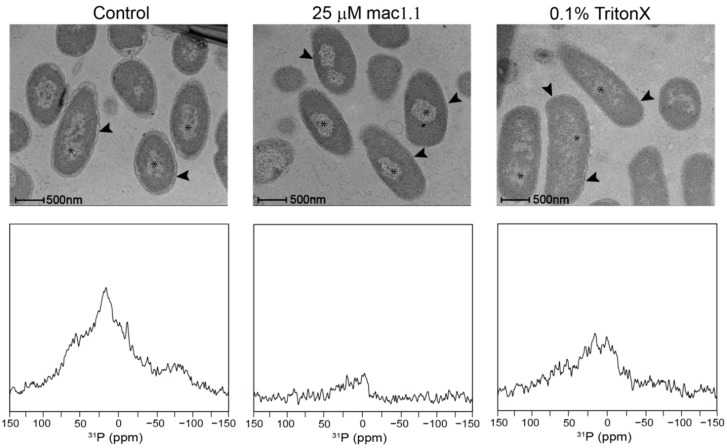
DNA morphology modulation and intermembrane collapse induced by Mac1.1: Upper panels show electron micrographs of *E. coli* (**left panel**), Mac1.1 treated (**middle panel**) and 0.1% triton-X treated (**right panel**). Asterisks indicates genomic DNA, arrow heads indicate the periplasmic space. *E. coli* were prepared as previously described for NMR experiments but washed in phosphate buffered saline (PBS) prior to preparation for transmission electron microscopy. Lower panel shows the comparable ^31^P CP NMR spectra performed at 30 °C.

The full effect of Mac1.1 on membrane integrity was difficult to determine by NMR given the spectral overlap both in the direct excitation and CP spectra. To further confirm the disrupting effect of Mac1.1 on *E. coli* membranes we utilized electron microscopy. *E. coli* cultured in the presence of 25 µM Mac1.1 showed a collapse of the periplasmic space with no distinction between the inner and outer membranes compared to untreated bacteria (Figure 6). This effect could be replicated in the presence of 0.1% Triton-X, indicating that the physiological disruption of the periplasmic space is similar to detergent treatment. Furthermore, Mac1.1 altered DNA morphology within the cytosolic space, causing the appearance of increased condensation compared to untreated *E. coli* while Triton-X had the opposite effect, dispersing the DNA (Figure 6). Comparison of ^31^P CP spectra of Mac1.1 and Triton-X treated cells revealed significant qualitative changes that were more pronounced in the case of Mac1.1. Signal intensity was severely reduced in both spectra due to cell death, impeding lineshape comparison. However, the spectra indicate that Triton-X has a minimal effect on the ^31^P CP lineshape, unlike the AMP (Figure 5C and Figure 6). Collectively, the data suggests that disruption of the ^31^P NA signal by Mac1.1 was not due to unraveling of genomic DNA and thus the AMP effect on NAs is distinct from its effect on membranes rather than as a consequence of membrane disruption.

## 3. Discussion

In-cell NMR provides a means for understanding the molecular effects of antimicrobial agents in a cellular context. While recent developments have focused on structure and folding of cytosolic proteins [21], little has been done to investigate the potential for using ^31^P as a molecular probe of cellular integrity. We have shown that phospholipids and NA can be readily distinguished in intact cells by ^31^P solid-state NMR and used effectively to probe the effects of antimicrobial agents on cellular integrity. Both ampicillin and Mac1.1 enhanced motional averaging of the ^31^P tensor of NAs, which may be partly attributed to possible ROS mediated damage of the NA backbone. Membrane integrity was also affected by both agents but due to spectral overlap could only be assessed through the intensity of the peak attributable to axially reorienting lipids.

The ^31^P spectral width observed in bacteria was greater than expected for live cells, particularly the presence of apparently rigid or immobilized molecules displaying a lineshape covering the full ^31^P CSA. Comparison with purified DNA corroborated the molecular identity of this signal and is also consistent with ^31^P lineshapes observed in viral particles in which the vast majority of ^31^P atoms can be attributed to NA in the form of viral genomic DNA or RNA [22]. Treatment of *E. coli* with either Mac1.1 or ampicillin resulted in similar lineshape alterations, with a reduction in signal intensity at the edges of the spectrum and a shift in spectral density towards 0 ppm, which collectively indicate increased dynamics of the ^31^P tensor of NA and confirmed by relaxation analysis of this signal in Mac1.1 treated bacteria. The partial recovery of this signal in ampicillin treated bacteria with thiourea needs further investigations and could involve ROS as ampicillin is widely known to induce ROS and is an important contributor to its effectiveness as an antimicrobial [19]. Since Mac1.1 is unable to create large pores [11], such as formed by cytolysins, it is unlikely that NAs are released from the intracellular space, causing the spectral changes observed. This was further confirmed by electron microscopy (EM) where Mac1.1 did not cause severe membrane disruption but rather increased DNA compaction. While the mechanism driving this morphological change in DNA is unclear, it is interesting to note that the CP spectra change in a similar way upon Mac1.1 and Triton-X treatment, showing different changes in DNA compaction by EM. If phosphodiester bond breakage occurred, then increased bond rotation of the phosphate would increase the isotropic nature of the signal and lead to reduced CP spectral intensity. In the absence of bond breakage, increased compaction would be expected to reduce T_1ρ_, resulting in greater signal intensities at short contact times, which was not observed in the semi-quantitative CP analysis (Figure 5D). Disruption of the DNA or RNA macromolecular structure, however, could also reduce the ^31^P powder pattern linewidth as would an increase in motion due to unraveled genomic DNA. Averaging of the ^31^P tensor would still be restricted by the phosphodiester bond dynamics and but may not change ^31^P dynamics to the same extent as expected in the case of bond breakage. Direct excitation spectra make it clear that this is a dynamic change, at least in the case of Mac1.1, in which spectral area is equivalent between treated and untreated cells. Breakage and fragmentation of NAs remains a possibility that requires further investigation using a combination of gel electrophoresis and mass spectrometry.

In the presence of thiourea, Mac1.1 treatment caused catastrophic cell death, resulting in almost complete destruction of the bacteria and dramatic loss of ^31^P NMR signal, preventing analysis. This was a powerful demonstration of the potential for combining sub-MIC concentrations of pore forming AMPs with other antimicrobial agents having different activity such that a normally protective anti-oxidant could become a powerful antimicrobial in the presence of AMPs. It is becoming increasingly clear that synergy between antimicrobial agents has the potential to raise the efficacy of existing antibiotics, even against strains that demonstrate resistance to frontline antibiotics like vancomycin [23,24]. This finding also highlights the potential for Mac1.1 to be used as a drug delivery system, particularly for other powerful antimicrobial agents that may exhibit poor membrane solubility and transport. 

The majority of AMPs have a strong affinity for lipid membranes, in particular negatively charged membranes, which confers specificity towards anionic prokaryotic membranes. Mac1.1 is a typical membrane-active peptide that has been shown to possess greater affinity for anionic membranes compared to neutral membranes and to form pores in bacterial membranes. A recent study showed that Mac1.1 alters *E. coli* membrane integrity well below the MIC and thus suggested that bacteria can cope differently with membrane damage [12], which would explain the difference in MIC between bacterial species. Both ampicillin and Mac1.1 destabilized membrane integrity, increasing the contribution of an isotropic component to the overall spectrum and a loss of signal from axially reorienting lipids. Disruption of the membrane by Mac1.1 was further visualized by electron microscopy with the appearance of a collapsed periplasmic space. 

Observation of phospholipid resonances was difficult due to signal overlap in both direct excitation and cross-polarization spectra, precluding detailed analysis of the effects of either antimicrobial agent on lipid dynamics and membrane structure. Furthermore, the observation of axially reorienting lipids in CP spectra in addition to direct excitation spectra inferred at a wide dynamic range of phospholipid dynamics in intact live bacteria. Lipids that form close interactions with membrane proteins would be expected to exhibit reduced dynamics and, therefore, able to be visualized in CP spectra at short contact times. The CSA of these powder patterns would reveal the dynamic range of these lipids, though this was not possible due to the dominance of the rigid NA signal. This range of dynamics in which phospholipid headgroups appear to exist in live cells potentially makes observation of lipids in cells difficult by ^31^P NMR as dynamic filters cannot be effectively used, at least in the absence of lipid specific labelling schemes [25]. However, disentanglement of heterogeneous dynamics in mixed lipid systems using MAS techniques [26] could be used in future to study live bacteria, providing bacteria integrity is preserved during MAS experiments.

## 4. Materials and Methods

### 4.1. Materials

Maculatin 1.1 (GLFGVLAKVAAHVVPAIAEHF-NH2; MW 2145) at >95% purity was made at the Bio21 Institute peptide synthesis facility (Melbourne, Australia). The peptide was washed in 5 mM HCl and lyophilized over-night to remove residual trifluoroacetic acid [27]. *Escherichia coli* total lipid extract was purchased from Avanti Polar Lipids (Alabaster, AL, USA) and used without further purification. Peptone, yeast extract, piperazine-*N*,*N*′-bis(2-ethanesulfonic acid) (PIPES) and sodium chloride were purchased from Sigma (Castle Hill, Australia).

### 4.2. Bacterial Growth

BL21 (DE3) *E. coli* was obtained from New England Biolabs (Ipswich, MA, USA.). Innoculation of an agar plate was made and incubated overnight at 37 °C. A single colony was transferred into a Luria broth (LB) culture flask and grown to an OD_600_ of 0.5 at 37 °C and under 250 rpm orbital shaking. Bacterial treatments were begun at OD_600_ of 0.5. Thiourea was used at a final concentration of 150 mM, ampicillin was used at 20 µg/mL and Mac1.1 at 10 and 25 µM where indicated. After the times indicated, the bacterial suspension was pelleted at 4000 rpm, washed twice with PIPES buffer (100 mM PIPES, 50 mM NaCl, pH 7.2). The supernatant was removed and the cell pellet was centrifuged down into a 4 mm Bruker NMR rotor (Billerica, MA, USA) at 1000 rpm.

### 4.3. Bacterial Survival

Survival of *E. coli* was determined by taking 5 µL of *E. coli* suspension from a packed rotor and performing serial dilutions in LB media, plated out onto agar and grown overnight at 37 °C. Colony forming units (CFU) were determined by counting the number of colonies. The percentage of remaining CFUs at various time points was calculated by: (number of CFU at 0 h)/(number of CFU at the indicated time points) [20]. 

### 4.4. E. coli Total Lipid Extract Sample Preparation

Multilamellar vesicles (MLVs) were formed using *E. coli* total lipid extract, which was resuspended in a large excess of Milli-Q water with or without Mac1.1, thoroughly homogenized and freeze-dried overnight. The dry powder was then resuspended in PIPES buffer at 65% (*w*/*w*) hydration and freeze-thawed three times prior to packing into a 4 mm MAS NMR rotor. 

### 4.5. Solid-State NMR Spectroscopy

^31^P NMR experiments were performed on a DNP-NMR 400 MHz Bruker Avance spectrometer at a frequency of 161.5 MHz. A 4 mm triple resonance probe was used in a double resonance mode. 62.5 kHz direct excitation pulse and 55 kHz Hartmann-Hahn ^1^H to ^31^P cross-polarisation (^31^P CP) experiments with 10% ramp on proton spin-lock were used under 31.25 kHz ^1^H SPINAL decoupling scheme. The recycle delay was obtained from ^1^H and ^31^P saturation recovery experiments, and set at 6 s (~5 T1). Typically, 512 scans and 3k scans were acquired for direct and 1.5 ms CP excitation experiments, respectively, except for the CP contact-time array and processed with 8k zero-filling and line-broadening from 50 to 500 Hz were used.

The CP spectra were integrated using the Topspin 3.5 software (Bruker, Billerica, MA, USA) and the spectrum area versus contact time experiments fitted according to [28]:

A(t) = A_0_ (1 − T_H-P_/T^H^_1ρ_) − 1 [exp(−t/T^H^_1ρ_) − exp(−t/T_H-P_)] (1)

where A(t) is the spectral area, A_0_ is proportional to an equilibrium Bloch magnetization, T_H-P_ is the CP time constant between ^1^H and ^31^P, and T^H^_1ρ_ is the ^1^H spin-lattice relaxation in the rotating frame. 

### 4.6. Electron Microscopy

Bacteria were grown in LB media until OD_600_ = 0.5 then treated with 25 µM Mac1.1 dissolved in water, water alone or 0.1% Triton-X for 30 min. The cells were then collected by centrifugation and washed twice with phosphate buffered saline prior to preparation for imaging. The samples were fixed with 1.5% formaldehyde, 1% glutaraldehyde in 0.1 M sodium cacodylate rinsed and post fixed in 1% osmium tetroxide in 0.15 M sodium cacodylate the serially dehydrate in ethanol and embedded in epoxy resin (Procure 812). Seventy nanometer sections were cut and stained with uranyl acetate and lead citrate. Samples were observed at 200 kV using a FEI Tecnai F30 (Hillsboro, OR, USA) equipped with a Gatan US1000 digital camera (Pleasanton, CA, USA).

## Abbreviations

NMR	Nuclear Magnetic Resonance
CP	Cross-Polarization
CSA	Chemical Shift Anisotropy
AMP	Antimicrobial Peptide
Mac1.1	Maculatin 1.1
DNA	Deoxyribonucleic Acid
CFU	Colony Forming Unit
